# On Superfœtation

**Published:** 1809-10

**Authors:** 


*Bath, August 29, 1809.*

---

### *To the Editors of the Medical and Physical Journal.*

Gentlemen,

In the present advanced state of medical knowledge, I should have thought that the doctrine of superfœtation was entirely excluded from the minds of all scientific men, had not my attention been called to the subject by perusing a Case inserted in your Journal for last month, and communicated by Mr. Fawell. It is not from the vanity of being a contributor to yours, or any other periodical publication, that I have undertaken the task of commenting upon that communication, but, because none of those Gentlemen, who by the extent of their medical knowledge and practice, are best able to explain the varying phœnomena of Nature, have favoured your readers with any comment thereon in your number for this month, month. I do not make pretentions to advance any thing novel.

Were I to say, that no such thing as superfœtation can take place in the human frame, perhaps, I should not trangress the bounds of truth. The knowledge of dogs bringing forth pups, evidently of different breeds, has not passed unnoticed by the advocates for the doctrine, but does not the construction of the uterus in many such animals, sufficiently account for that circumstance? A bitch may have been lined by half a dozen different dogs, and bring forth a pup evidently of each breed; this may, at first sight, look like superfœtation, *or rather super-conception*, which so far may be allowed, yet, at the time of birth, every pup is perfectly matured, and generally in a living state. Now, this would not be the case, if the different impregnations had taken place at much distance of time from each other; and we never hear of pups being produced of different ages, without the evident existence of disease in those pups.

I recollect, some time ago, reading of an animal said to be capable of having fœtuses of different ages in utero at the same time; but from not being at that time in the habit of noting down what appeared to me in the course of my reading worthy of observation, unfortunately I am not able at present, to call to mind the work in which the subject was mentioned, nor the name of the animal. However, allowing the fact, are we from thence to conclude, that the same may take place in the human uterus? I conceive, that if it is not altogether impossible, there is the greatest improbability of it. It is true, that authors have not been wanting in the recital of such cases; but in those which have fallen within the sphere of my reading, upon consideration, I think they may be accounted for upon different principles than those of superfœtation.

When the natural economy of mankind, and the changes which take place in every healthy human uterus soon after conception are considered, the possibility of a further impregnation, at a period so distant as three or four months, (which must have happened in the case before us) is entirely precluded.

The advocates for the doctrine, bring forward the story of a woman in North Carolina, who had a white and a black child at the same birth. She was supposed to have had connection with a black man first, and afterwards with her husband, who was a white. In the account I have read, we are not informed whether both were living living or dead. I have no reason to doubt the authenticity of the story, nor indeed am I at all inclined to do so, for, if I am not mistaken, it is allowed, by those who have paid attention to the subject of human impregnation, that the os uteri is not closed immediately after conception has taken place, but that some time is necessary for that change to be effected. In this manner, the story may be sufficiently elucidated, and the wonder diminished; but, though I should allow thus far the doctrine of superfœtation, (or more properly that of super-conception) yet, I cannot for a moment suppose, that after the os uteri has been perfectly closed (which it must be after conception) it will be at any time during a state of utero-gestation in such a state as to favour the admission of any thing; and I am not one of those, who think that impregnation can take place any other way than by the actual admission of the semen into the cavity of the uterus.

In every other case that I have read, the fœtus or child which has appeared to be youngest, has been dead at the time of its birth; but the accounts of the apparent period that has elapsed since that event took place, do not so directly coincide. Mr. F. in the case in question, gives it as his opinion, that the child had been dead a few days, and I should think founds that opinion upon the circumstance of putrefaction not having taken place. But will it authorize the opinion? There are such various ways to account for putrefaction not having taken place, that I am inclined to think not. The presence of air is supposed to be in a great measure, if not absolutely, necessary to occasion that event. Then, if no air had been admitted, would putrefaction have taken place? I am aware of the liquor amnii, when discharged in the regular course of labour, being sometimes so fœtid as to render it almost impossible to remain in the room with the patient. This is mentioned as a sign of the child being dead, and is no doubt occasioned by putrefaction. Here then, putrefaction has taken place in utero, but has not air found admission in these cases at some period of uterogestation? I think that it has, for upon the death of the child taking place, the ovum suffers a partial or more general detachment, which is proved by a slighter or larger sanious discharge from the uterus coming on then, and surely the admission of so subtle a fluid as air might take place at that time. Yet, in the case before us, no putrefaction had taken place, nor were the waters offensive. This I account for by its being a case of twins, though one one child was deprived of all vital principles, yet the other still enjoyed them fully. Had air been admitted into this uterus, would not both have been dead?

It may be that the child had not been long dead, the vital powers may not have been extinguished above five or six days. If so, it may be asked, how will you account for the smallness of its size? I answer, by disease having taken place in the child. We are not without instances of children being affected with various diseases there. A child has been born loaded with the small-pox; another, with a stone in the bladder. We are daily in the habit of seeing children affected with disease, so far from increasing in size, apparently decreasing; and this event is equally likely to take place in utero as the disease.

Again, if the doctrine of superfœtation be allowed, will it be unreasonable to suppose, by the same laws through which this is effected, that the fœtus last conceived would not be expelled from the uterus, till it had completed its full period of uterogestation, notwithstanding the first born may be at its own proper period? This we know would be impossible to be the case, since, if the action of the uterus be brought on by any cause, or at any period of pregnancy, it will not cease till the whole of its contents are evacuated.

My opinion of the case is simply this; the woman has conceived twins, and at a certain period of pregnancy, one of the fœtuses has died. That this might take place, is very possible, and, nevertheless, the other child may not be in the least affected with the change that has happened to its fellow, since there is no communication whatever between the fœtuses in utero, each receiving its support from its own placenta, and being envelloped in its own membranes.

LEODINENSIS.

*August* 8, 1809.

